# Assessment of Radiation-Induced Optic Neuropathy in a Multi-Institutional Cohort of Chordoma and Chondrosarcoma Patients Treated with Proton Therapy

**DOI:** 10.3390/cancers13215327

**Published:** 2021-10-23

**Authors:** Andreas Köthe, Loïc Feuvret, Damien Charles Weber, Sairos Safai, Antony John Lomax, Giovanni Fattori

**Affiliations:** 1 Center for Proton Therapy, Paul Scherrer Institute, 5232 Villigen, Switzerland; damien.weber@psi.ch (D.C.W.); sairos.safai@psi.ch (S.S.); tony.lomax@psi.ch (A.J.L.); giovanni.fattori@psi.ch (G.F.); 2 Department of Physics, ETH Zürich, 8093 Zürich, Switzerland; 3 Center for Proton Therapy, Institut Curie, 91400 Orsay, France; loic.feuvret@aphp.fr; 4 Department of Radiation Oncology, AP-HP, Hôpitaux Universitaires La Salpêtrière Charles Foix, Sorbonne Université, 75013 Paris, France; 5 Department of Radiation Oncology, University Hospital Zürich, 8091 Zürich, Switzerland; 6 Department of Radiation Oncology, University Hospital Bern, 3010 Bern, Switzerland

**Keywords:** proton therapy, RION, NTCP, optic toxicity, skull base, chordoma, chondrosarcoma, risk factors, normal tissue effects

## Abstract

**Simple Summary:**

Proton therapy is an effective therapeutic option for the treatment of skull-base tumors that require high radiation doses to be controlled. On rare occasions, patients suffer from radiation-induced optic neuropathy (RION) to the detriment of their post-treatment quality-of-life. We have collected multi-institutional data of 289 skull-base patients having received high doses to the optic apparatus from proton therapy or proton–photon mixed treatments and have observed a RION incidence rate (all grades) of 4.2% (12). We have furthermore confirmed older age and hypertension as risk factors for the onset of this side effect, with tumor involvement or its proximity to the optic apparatus and repeated surgical procedures showing moderate association. Our findings were consolidated into a NTCP model that can support pre-treatment patient segmentation into risk groups and the planning of necessary treatment countermeasures. However, further data and validation are necessary to confirm validity of the model.

**Abstract:**

Radiation-induced optic neuropathy (RION) is a rare side effect following radiation therapy involving the optic structures whose onset is, due to the low amount of available data, challenging to predict. We have analyzed a multi-institutional cohort including 289 skull-base cancer patients treated with proton therapy who all received >45 Gy_RBE_ to the optic apparatus. An overall incidence rate of 4.2% (12) was observed, with chordoma patients being at higher risk (5.8%) than chondrosarcoma patients (3.2%). Older age and arterial hypertension, tumor involvement, and repeated surgeries (>3) were found to be associated with RION. Based on bootstrapping and cross-validation, a NTCP model based on age and hypertension was determined to be the most robust, showing good classification ability (AUC-ROC 0.77) and calibration on our dataset. We suggest the application of this model with a threshold of 6% to segment patients into low and high-risk groups before treatment planning. However, further data and external validation are warranted before clinical application.

## 1. Introduction

Chordomas and chondrosarcomas are slowly growing malignant tumors of the skull-base which, due to their relatively high radioresistance and challenging location for surgery making gross tumor removal unlikely, require high doses of adjuvant radiation to be effectively controlled [1]. Consequently, nearby organs-at-risk (OARs), such as the optic structures, can be exposed to high doses and can therefore be associated with a potential risk of late toxicity. A rare, but detrimental side effect is radiation-induced optic neuropathy (RION) [2], which is caused by damage to the optic nerves or chiasma which, in the most severe cases, can lead to uni- or bilateral blindness. Proton therapy is particularly of interest for the treatment of such tumors due to its ability to deliver high radiation doses whilst sparing critical OARs surrounding the target, such as the temporal lobes, nerve tissue, or the optic apparatus [3,4]. Despite that, incidences of RION have been reported in clinical studies, although the low numbers challenge the development of predictive models [5,6]. While normal tissue complication probability (NTCP) models for RION can be found in the literature [5,7,8], data for treatments in the skull-base are extremely scarce, with a distinct lack of validated models. 

RION typically appears several months or a few years after treatment [9] and therefore requires sufficient follow-up to be detected. In addition, clinical risk factors such as age or hypertension have been demonstrated to also play a role in RION onset and have recently been integrated into NTCP models [5,10]. This however requires datasets with a great level of detail, including dosimetry, clinical patient characteristics, and sufficient follow-up, which, in conjunction with the low incidence rate of RION, is challenging to acquire. 

In an attempt to advance the understanding of the causes of RION, we have collected and analyzed multi-institutional data from skull-base patients treated with proton therapy at two European facilities. Our goal was to report the incidence rates of RION, to determine and validate risk factors, and to propose a NTCP model that could ultimately help personalize radiotherapy treatment planning for skull base indications by accounting for the individual patient risk of RION. 

## 2. Materials and Methods

### 2.1. Patient Data

Two independent consecutive cohorts were collected retrospectively from the Center for Proton Therapy (CPT) at the Paul Scherrer Institute (PSI), Villigen, Switzerland, and the Institut Curie, Centre de Protonthérapie (CPO) in Orsay, France. Only skull-base adult patients with no history of chemotherapy and who had received at least 45 Gy_RBE_ to the optic apparatus (optic chiasm and the left and right optic nerves) were included. A comprehensive overview of patient and treatment characteristics as well as a statistical comparison between the institutes is given in Table 1. The complete cohort consisted of 289 individual records, out of which 101 (35%) were skull-base chordomas and 188 (65%) were chondrosarcomas. Half of the patients (*n* = 143) were treated at PSI with pencil beam-scanned (PBS) proton therapy, and the remaining (*n* = 146) patients were treated at CPO using either passive scattered (PS) protons alone (*n* = 52) or a combination of photon and PS protons (*n* = 94). All of the patients from CPO were treated for chondrosarcomas while the PSI cohort included both chordomas (101, 71%) and chondrosarcomas (42, 29%). Standard fractionations (1.8-2 Gy_RBE_/fraction) were used across the cohort, with median prescription doses of 74 Gy_RBE_ for chordomas (range, 68–74) and 70 Gy_RBE_ (range, 67–76) for chondrosarcomas. Treatments at PSI were planned using an in-house developed treatment planning system [11] that has been in clinical use since 1996. Treatment plans between 1996 and 2008 at CPO were generated using the ISIS 3D treatment planning system (Technology Diffusion). For later treatments, the ISOgray treatment planning system (DOSIsoft, Cachan, France) was employed. A fixed RBE of 1.1 was assumed for all proton treatments. Patients in the cohort were treated between 1996 and 2019 and had a median follow-up of 5.6 years (range 0.5–16 years). RION toxicity was scored according to CTCAE v5.0 and confirmed by MR imaging. 

### 2.2. Dataset Analysis and NTCP Modeling

To develop an NTCP model to predict the risk for RION of any grade, the predictive capacity of a comprehensive range of clinical and dosimetric variables has been evaluated. Clinical variables included tumor type (chordoma or chondrosarcoma), patient age, the presence of arterial hypertension (high blood pressure (HBP) associated with a resting blood pressure above 140/90 mmHg), sex, the number of surgeries prior to radiotherapy, and GTV volume. Dosimetrically, eight parameters were included in our analysis. The total prescription dose, the total proton dose, and the total photon dose contributions we also included (for combined treatments only). For the optic structures (chiasm, right and left optic nerves) the minimum (D_min_), mean (D_mean_), and maximum (D_max_) doses as well as D_1%_ and D_99%_ (doses given to 1% and 99% of the volume, respectively) were also considered. The assumed model type was a logistic regression model of the form:

(1) $$NTCP_{RION}=\frac{1}{1+e^{-\beta_{x}}}\;\mathrm{with},$$

(2) $$\beta_{x}=\beta_{0}+\beta_{1}x_{1}+\beta_{2}x_{2}+\cdots +\beta_{n}x_{n}$$

with $\beta_{0}$ the offset and $\beta_{i}$ the parameters associated with the patient variables $x_{i}$ (e.g., $\beta_{Age}$).

Prior to modeling, univariable correlation to RION incidence was assessed for all variables using a logistic regression fit for continuous variables and a Chi square test for the categorical ones. In the following, strongly cross-correlated variables were eliminated by assessing their Spearman ranked correlation coefficient [12] (SRCC). If two variables were strongly cross-correlated (SRCC > 0.8), the variable with the lower SRCC (to RION incidence) was eliminated from the modeling process. Furthermore, parameter robustness was challenged by bootstrapping analysis [13] using 1000 sub-samples of the entire dataset with replacement. The least absolute shrinkage and selection operator (LASSO) method was applied to each bootstrap sample and based on the analysis of each parameter’s selection frequency, the most robust parameters were considered for further analysis. The minimal regularization parameter was selected so that it allowed for a maximum of four model parameters for each LASSO-based model. All of the combinations of highly ranking features were then evaluated with extensive cross-validation (CV) analysis to counteract overfitting. Leave-one-out, 5-fold, and 10-fold CV were performed with a random re-ordering of the samples in 10 repetitions. In case of missing data, complete case analysis was used. Performance was evaluated using Akaike [14] (AIC) and Bayes [15] (BIC) information criteria, area under the curve of the receiver operating characteristics (AUC-ROC), cross-entropy (CE), defined as the negative ratio between log-likelihood and the number of samples, and a Hosmer–Lemeshow (HL) test statistic.

Finally, instead of simply taking the single best-fit parameters for our dataset, the parameter values were determined by penalized regression (LASSO). The regularization parameter was selected based on 10-fold cross-validation, minimizing the deviance. The fitting process was repeated 100 times, and the median model parameter values selected to minimize bias from a certain patient order from cross-validation. Confidence intervals were determined on parameter distributions obtained from 1000 bootstrap samples and un-penalized logistic regression fits. In all cases, a statistical significance threshold of 0.05 was used and all statistical analysis was performed in MATLAB (R2018b, The Mathworks, Natick, CA, USA). 

## 3. Results

### 3.1. RION Incidence

Twelve (4.2%) patients suffered from RION following radiotherapy. Three (25%) developed a grade 1, one a grade 2 (8.3%), three a grade 3 (25.0%), and five (41.7%) a grade 4 complication. For the majority (*n* = 9; 75%) of patients with RION, only one optic structure was affected. One patient however presented RION in the left and right optic nerves, and two patients developed a complication in both optic nerves as well as in the chiasm. The maximum dose to the optic apparatus in patients with RION was between 56 and 63 Gy_RBE_ (Median 59). The RION incidence rate was higher in chordoma patients (6/101-5.9%, data from PSI cohort only) than in chondrosarcoma patients (6/188-3.2%). For the latter indication, comparable incidence rates were found for both the PSI and CPO patient cohorts (1/42, 2.4% and 5/146, 3.4%, respectively).

### 3.2. Univariable Correlation to RION

Out of all of the considered variables, three were significantly associated with RION in the univariable correlation analysis: age (*p*-value = 0.008), arterial hypertension (*p*-value < 0.001), and more than three surgeries prior to radiotherapy (*p*-value = 0.038). A detailed overview of the variables and their *p*-values is reported in Table 2. 

Statistical differences between the institutes were detected for (see Table 1) the type of tumor, the maximum dose to the optic structures, the gross tumor volume (GTV), prescription doses, the number of surgeries, tumor involvement in the optic apparatus as well as the treatment modality (protons vs. mixed regimen and PBS vs. PS protons). All of these are directly linked to the fact that the CPO cohort entirely consists of chondrosarcoma patients and were treated with mixed regimen or PS proton therapy. No significant differences were detected for more general cohort characteristics, such as age, sex, hypertension, or RION incidence. 

### 3.3. Elimination of Cross-Correlation and Bootstrapping

**Figure 1:**
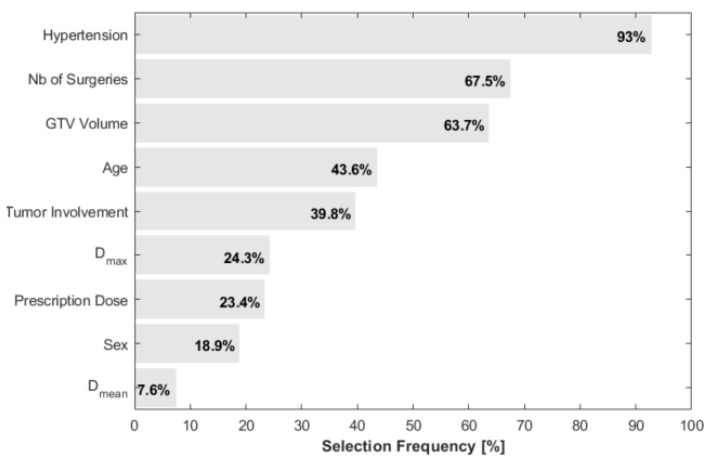
LASSO-based parameter selection frequency over 1000 bootstrap samples.

Strong collinearity (SRCC > 0.8) was detected between photon doses and proton doses to the target. Further correlation was observed between D_99_, mean and minimum doses in the optic structures as well as between D_1_ and the maximum dose. Eliminating the cross-correlated variables with the lowest SRCC to RION incidence resulted in nine remaining variables being used for the LASSO regression in the bootstrap analysis. These were age, hypertension, tumor involvement, sex, the number of surgeries prior to radiotherapy, GTV volume, prescription dose, and the mean and maximum doses to the optic apparatus. As a result, from the bootstrapping parameter selection, this set of variables was furthermore narrowed down to the most robust variables in descending order of selection frequency (Figure 1): hypertension (93%), the number of surgeries (68%), GTV volume (64%), age (44%), and tumor involvement (40%). Note that the highest ranking dosimetric parameter was the maximum dose, with a frequency of 24%.

### 3.4. Multi-Parametric Model Selection and Cross-Validation Analysis

**Figure 2:**
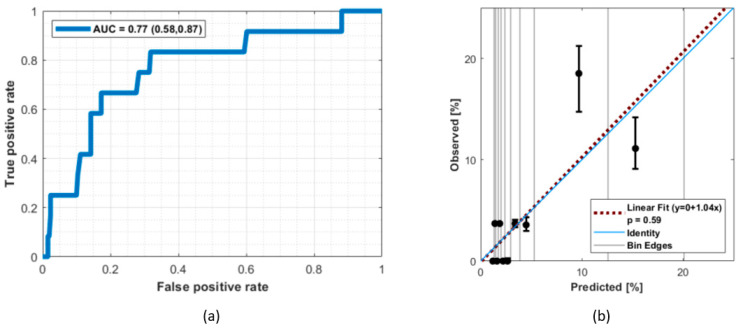
(**a**) The receiver operating characteristic curve for the bivariable logistic regression model using age and hypertension as input parameters with an area under the curve of 0.77 (0.58,0.87–90% confidence intervals). (**b**) Model calibration. A Hosmer–Lemeshow *p*-value of 0.59 and a model slope from the linear fit of 1.04 confirm that the model is calibrated. The black circles represent the observed proportion of RION-positive patients in each specific bin of patients (with 90% confidence intervals) plotted against the predicted average NTCP for the same patient set.

**Figure 3:**
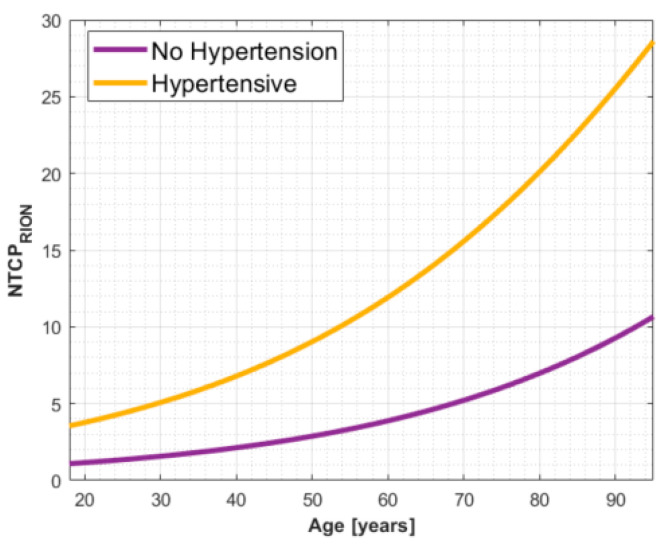
Visual representation of the final NTCP model formulation based on hypertension and age. Hypertensive as well as older patients are at larger risk for RION. This model applies to patients that receive at least 45 Gy_RBE_ to the optic apparatus.

From the bootstrapping and cross-validation results, the bivariable logistic regression model built on age and hypertension performed the best, with a mean AUC-ROC across all cross-validation runs of 0.68. A detailed overview of the cross-validation results is reported in Appendix A. The final model parameters (with 90% confidence intervals (CI)) fitted on the entire dataset are $\beta_{offset}=-5.07\;\left(-8.7,-3.4\right),\;\beta_{Age}=0.031\;\left(-0.01,0.09\right)\;\mathrm{and}\;\beta_{HBP}=1.21\left(0.04,\;2.84\right)$, resulting in an AUC-ROC (90% CI-Figure 2a) of 0.77 (0.58, 0.87) and good calibration of the combined cohort, with a HL *p*-value of 0.59. The calibration curve depicted in Figure 2b shows that the model is relatively well-calibrated on our dataset. A visual representation of the final NTCP model for RION is given in Figure 3.

### 3.5. A Threshold for Patient Segmentation into Risk Groups

**Figure 4:**
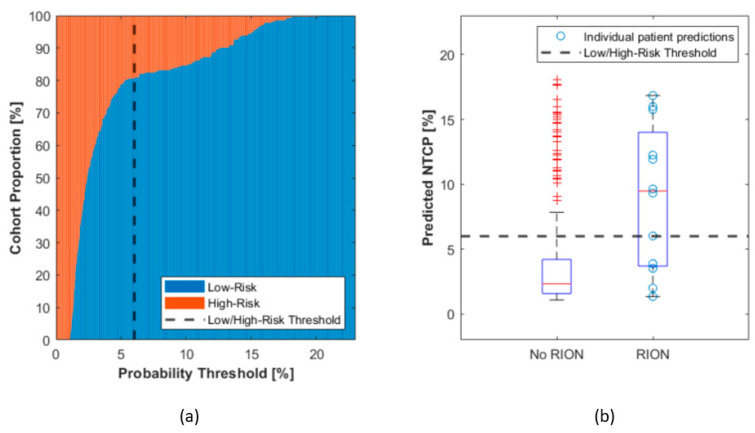
Threshold selection to segment patients into high- and low-risk groups. (**a**) The proportion of patients in each group is plotted for different thresholds. A threshold of 6% is proposed for patient classification into risk groups. (**b**) Boxplots for the predicted NTCPs for all patients in our cohort, and cases with and without RION reporting are analyzed individually. The selected threshold of 6% would permit high specificity (83%) and good sensitivity (67%) in our data.

Based on risk estimations provided by the NTCP model, patients can be categorized into low- or high-risk groups for RION. Proportions of the cohort with low or high-risk as a function of NTCP probability threshold are shown in Figure 4a. We propose the setting of a threshold to identify high-risk subjects at the level of 6% (dashed line–Figure 4). As such, one out of five patients from our cohort would be considered as high risk based on this derived model and threshold (Figure 4a). Furthermore, this threshold allows two thirds of patients with RION incidence to be placed in the high-risk group (sensitivity 67%) and provides good specificity (83%) in our cohort (Figure 4b).

## 4. Discussion

We analysed a large multi-institutional cohort of skull-base proton therapy patients, all of whom received at least 45 Gy_RBE_ to the optic apparatus and reported an overall RION incidence rate of 4.2%. Older age, hypertension as well as repeated surgeries (>3) prior to radiotherapy were identified as significant risk factors. In addition, tumor involvement in the optic apparatus and the GTV volume showed a trend towards contributing to the onset of this side effect. From these factors, a NTCP model was derived integrating the risk factors of age and hypertension to estimate the probability of RION, which could be used for more precise patient counseling as well as for patient segmentation into risk groups prior to treatment planning. 

The database, which features a long follow-up time (median > 5 years), is particularly suited for RION studies, a toxicity that typically appears a few months up to years after radiotherapy. However, to reach large numbers, we compromised on the homogeneity of the indications across the institutions, combining chondrosarcoma patients from CPO with the PSI dataset, which also included chordomas. While the overall RION incidence was found to be higher (5.9%) for chordomas, the incidence for chondrosarcoma patients was similar between the PSI (2.4%) and CPO (3.4%) cohorts. Furthermore, all patients from PSI were treated with PBS proton therapy, whereas CPO used scattered protons, sometimes in combination with photons. In agreement with Ali et al. [9], no significant correlation between the treatment modality (photon vs. proton) and the onset of RION, which could have otherwise jeopardized use of the combined dataset, was observed in our data. Such controlled variability, in addition to the individual institution practices and treatment planning experience, is expected to contribute to the robustness of fitted parameters and add to the general value of the proposed NTCP model.

RION is a rare side effect whose causes are, due to the scarcity of clinical reporting available, poorly understood. Indeed, among the considered 289 skull-base patients in our cohort, only 12 events were observed. This is in agreement with published data on head, neck, and brain cohorts. Demizu et al. [4] reported an incidence of 6% (7/120) following proton therapy, a slightly lower rate of 2.8% (1/36) was documented by Weber et al. [16], and Urie et al. [17] estimated an incidence rate of 5% at the 70 Gy dose level. Li et al. [5] showed an incidence rate of 3.3% (17/514), which is slightly lower than the rate seen in our cohort, which is likely due to the lower dose inclusion criterion of 30 Gy_RBE_ instead of our 45 Gy_RBE_ dose. Moiseenko et al. [8] reported a rate of 11% (19/172). However, this higher rate might be biased by the outdated treatment techniques since the patients who were included in that cohort were treated between 1964 and 2000. Furthermore, we observed a lower incidence rate in chondrosarcomas (3.2%) compared to chordoma patients (5.9%). This is in agreement with older age being a risk factor, as the chondrosarcoma group was younger than the chordoma group (mean 43 vs. 48.3 years, respectively). Despite not being statistically significant in our cohort, the higher prescription dose (74 Gy_RBE_) to treat chordoma as opposed to the 70 Gy_RBE_ dose to treat chondrosarcoma could have also contributed. Finally, this study confirms the validity of the constraints applied to the optic apparatus [18], which, despite an inclusion criterion of a higher dose, resulted in few cases of RION. 

Developing a NTCP model from a very imbalanced dataset with only 12 cases of RION grade ≥ 1 is challenging. While we cannot exclude statistical artefacts affecting model robustness and generalizability, we have attempted to minimize this risk by making extensive use of bootstrapping, cross-validation, and penalized regression techniques. Although additional clinical parameters could also play a role in RION, for example chemotherapy [2], diabetes mellitus [4], or genetic pre-disposition, such data were not available in our cohorts and have only been inconsistently reported as risk factors for RION [6]. 

Older age, hypertension, and tumor involvement with the optic apparatus have been significantly associated with the onset of toxicity, confirming previous reports for both photon and proton-based radiotherapy [5,6,9,19,20]. In contrast to previous reporting [5,10], and although both sexes were equally represented (152F /137M) in our cohort, sex was not significant in our dataset. In addition, we found that the number of surgeries (>3) prior to therapy was associated with RION in the univariable correlation analysis. However, despite being reported elsewhere [9], this correlation was weak in our analysis. Treating the number of surgeries as a continuous variable did not result in a significant correlation, as only 12 patients underwent more than 3 surgeries, two of whom suffered RION afterwards. A patient cohort with a higher number of surgeries would be needed for further confirmation of the significance of this as a risk factor for RION. Interestingly, and in agreement with previous studies from our group [10], no dose metric was found to be significantly associated with RION other than the inclusion criterion of at least 45 Gy_RBE_ being planned to the optic apparatus. While this cut-off has been set arbitrarily, there is general agreement that high doses are a prerequisite for the onset of RION [4,9,21], and toxicity has rarely been reported in clinical studies for doses below 45Gy [6]. 

In a previous publication, we introduced two RION NTCP models [10] from a cohort of skull-base and head and neck cancer patients treated with PBS proton therapy based on age, tumor involvement, and either sex or hypertension, respectively. Although the present cohort is limited to chordoma and chondrosarcoma, and hence the inhomogeneity of populations, prevents its use for the external validation of the models mentioned above, it is nevertheless possible to confirm some of the hypothesis and risk factors discussed in our previous study. In particular, since no other dose metric was found to influence the risk for RION other than the inclusion criterion, our speculation that above a certain level, clinical characteristics such as age, hypertension, or tumor proximity determine the actual risk for this side effect is supported by this multi-institutional analysis. Moreover, the reassessment of statistical power for sex, or the lack of thereof, indicates that its significance as a risk factor as reported in [10] might have been a statistical anomaly with limited generality.

In principle, the NTCP model in this work could be used to stratify patients into RION-specific high- and low-risk groups prior to the commencement of therapy. As such, and based on our data, we propose a NTCP threshold for high-risk patients at 6%. This would result in 20% of all patients being eligible for the model application to be considered at significant risk, even though only 15% of these patients actually presented with RION. While we are aware that the threshold selection is arbitrary, we proposed 6%, which could even be considered an “acceptable” level of toxicity following radiation therapy. Any threshold below 5% was considered unsuitable due to the high sensitivity of the population proportion (low vs. high risk—Figure 4a) against small threshold variations. Furthermore, this threshold optimized sensitivity as well as specificity in our dataset, resulting in a 20/80% classification ratio between high and low risk patients. We do however encourage each treatment facility to assess their priority on classification sensitivity or specificity and adjust the threshold as appropriate. For example, it might be of interest to improve sensitivity by increasing the threshold in order to include a higher proportion of patients in the high-risk group that will ultimately be affected by RION. This patient stratification could ultimately be applied to improve patient awareness through the communication of their specific risk for RION based on their individual risk factors. Moreover, high-risk patients could be considered more thoroughly for treatment modalities with higher potential to spare OARs, such as proton therapy. However, despite being a devastating side effect at higher grades, RION still only seldomly appears and there might not be enough evidence to f.i. compromise on target coverage, hence prejudiced local control, to decrease dose to the optic apparatus, especially for chordoma indications. In addition, other toxicities, such as brain necroses or radiation-induced pituitary disorders need to be considered when planning a radiotherapy treatment. Furthermore, there is no general consensus on the prescription dose for chondrosarcoma, which is typically between 65 and 70 Gy. On the condition of more reliable data, a reduction in the prescription dose for this indication could be considered to maintain high tumor control while reducing late toxicities. Although no specific dosimetry risk factor emerged from our study, the maximum dose to the optic apparatus has been previously reported to be correlated to RION [4] (and was the most prominent dosimetric factor in our cohort), and therefore, in the treatment planning process, focus could be put towards decreasing the maximum dose to the optic structures for high-risk subjects. 

Finally, since no significant difference was found between proton only treatments and combined photon–proton fractions, a result also reported in [9], the proposed NTCP model and risk classification strategy could possibly be extended to conventional X-ray therapy. This could not only be used to support patient selection between modalities (proton vs. photon) but also photon treatment data could be used for model update and external validation.

## 5. Conclusions

We reported the onset of RION in 4.2% of skull-base tumor patients planned with high dose to the optic apparatus. Based on our multi-institutional data analysis, the incidence was found to be lower in chondrosarcoma patients (3.2%) compared to chordoma patients (5.9%). We confirmed older age and arterial hypertension as major risk factors, with a higher number of surgeries, a larger GTV volume, and tumor involvement in the optic structures being moderately associated with RION incidence. A NTCP model was proposed, permitting the assessment of individual patient risk for RION prior to radiotherapy and recommend treatment plan optimization for patients who are at high risk (>6%). However, data from other institutions need to be pooled and integrated into the NTCP model for external validation before clinical implementation. 

## Figures and Tables

**Table 1:** Patient characteristics for the combined cohort and the parts from both institutions (PSI, CPO). X (Y-Z) indicates the median (X) and the range for this variable (Y-Z). A statistical comparison between the parts of each cohort was performed. A *p*-value is computed based on a Wilcoxon rank sum test for continuous and a Fisher’s exact test for categorical variables. *p*-values below 0.05 (bold) are considered to show significant differences between the institute data.

Property	Total	PSI	CPO	*p*-Value CPO vs. PSI
Number of patients	289 (100%)	143 (49%)	146 (51%)	-
Age (years)	43 (18–80)	45 (18–77)	44 (18–80)	0.17
Tumor Type	Chordoma (101, 35.0%), Chondrosarcoma (188, 65.0%)	Chordoma (101, 70.6%), Chondrosarcoma (42, 29.4%)	Chondrosarcoma (146, 100%)	**<0.001**
Minimum dose to optic apparatus (GyRBE)	2 (0, 50.5)	0.9 (0.1,34.4)	2.7 (0, 50.5)	0.88
Maximum dose to optic apparatus (GyRBE)	59.6 (45,76)	60.0 (45.6,76)	59.3 (45,72.4)	**0.03**
GTV Volume (cc)	27.2 (0.2–130)	34.9 (2.3–130)	19.6 (0.2–109)	**<0.001**
Treatment doses (GyRBE)	71.2 (67–76)	72.7 (68–76)	70.2 (70–70.2)	**<0.001**
Follow-Up (months)	73.8 (5–197)	75.1 (11.83–183.6)	72.5 (5–197)	0.11
Treatment Year	1996–2019	1999–2013	1996–2019	-
Number of Surgeries	1.6 (1–8)	1.9 (1–8)	1.3 (1–4)	**<0.001**
RION cases (grade ≥ 1)	12 (4.2%)	7 (4.9%)	5 (3.4%)	0.57
Sex	152F/137M	67F/76M	85F/61M	0.06
Hypertension (yes/no/no data)	52/221/16	30/98/15	22/123/1	0.09
Tumor Involvement in the Optic Apparatus (yes/no/no data)	112/174/3	42/101/0	70/73/3	**0.001**
Protons Only	195 (67%)	143 (100%)	52 (35.6%)	**<0.001**
Pencil Beam Scanning	143 (49%)	143 (100%)	0 (0%)	**<0.001**

**Table 2:** Univariable correlation between RION incidence and a range of variables. Statistically significant variables are highlighted in bold.

Parameter	*p*-Value
**Age**	**0.008**
Tumor (Chordoma/Chondrosarcoma)	0.264
**Hypertension**	**<0.001**
Tumor Involvement	0.432
Sex	0.684
Number of surgeries	0.468
Number of surgeries > 1	0.932
Number of surgeries > 2	0.273
**Number of surgeries > 3**	**0.038**
Number of surgeries > 4	0.577
GTV Volume	0.731
Prescription Dose	0.167
Photon Dose	0.419
Proton Dose	0.321
D_mean_	0.773
D_max_	0.131
D_min_	0.594
D_1_	0.150
D_99_	0.663

## Data Availability

The data presented in this study are available upon request from the corresponding author. The data are not publicly available due to sensitive patient information.

## Appendix A

Appendix A: Mean AUC-ROC, cross-entropy (CE), Hosmer-Lemeshow (HL) *p*-values, AIC and BIC metrics for 10 repetitions of leave-one-out,5- and 10-fold cross-validation on the dataset for the indicated models. The best performing model was found to be based on age and hypertension. HBP: Hypertension, TI: Tumor involvement, GTV: GTV Volume, Surg: Number of surgeries prior to radiotherapy.

**Table A1:** The detailed overview of the cross-validation results.

	Mean over All k-Fold (LOO,5,10) with 10 Repetitions				
	Mean AUC	Mean CE	Mean HL *p*-Value	AIC	BIC
Age	0.68	0.17	0.45	100.4	107.8
HBP	0.50	0.17	0.00	97.3	104.5
TI	0.09	0.18	0.00	106.5	113.8
Surg	0.08	0.18	0.00	106.0	113.4
GTV	0.08	0.19	0.00	112.1	119.4
Age HBP	0.68	0.17	0.14	100.5	111.3
Age TI	0.65	0.17	0.33	105.8	116.8
Age Surg	0.65	0.17	0.55	104.3	115.3
Age GTV	0.62	0.19	0.10	112.3	123.2
HBP TI	0.58	0.18	0.00	101.3	112.1
HBP Surg	0.53	0.17	0.00	101.1	111.9
HBP GTV	0.51	0.21	0.00	113.9	124.6
TI Surg	0.07	0.18	0.00	107.8	118.7
TI GTV	0.08	0.19	0.00	110.7	121.5
Surg GTV	0.07	0.19	0.00	111.9	122.7
Age HBP TI	0.65	0.18	0.15	105.7	120.1
Age HBP Surg	0.63	0.18	0.05	104.0	118.5
Age HBP GTV	0.56	0.22	0.03	120.8	135.0
Age TI Surg	0.61	0.18	0.47	108.2	122.8
Age TI GTV	0.61	0.19	0.13	114.9	129.4
Age Surg GTV	0.62	0.19	0.13	112.4	126.9
HBP TI Surg	0.52	0.18	0.00	103.1	117.5
HBP TI GTV	0.50	0.21	0.00	115.9	130.2
HBP Surg GTV	0.51	0.21	0.00	115.9	130.2
TI Surg GTV	0.07	0.19	0.00	112.4	126.9
Age HBP TI Surg	0.59	0.18	0.05	108.4	126.3
Age HBP TI GTV	0.55	0.21	0.02	120.2	138.0
Age HBP Surg GTV	0.58	0.22	0.02	122.9	140.7
Age TI Surg GTV	0.59	0.19	0.09	114.0	132.1
HBP TI Surg GTV	0.50	0.21	0.00	116.8	134.5
Age HBP TI Surg GTV	0.56	0.20	0.03	118.1	139.4
